# Supplementary material for: A whole-animal phenotypic drug screen identifies suppressors of atherogenic lipoproteins
Source: eLife. 2026 Aug 27;14:RP105314. doi: 10.7554/eLife.105314 (PMC13521566; doi:10.7554/eLife.105314)
Supplement: Supplementary file 2 [file elife-105314-code1.zip › Figure_3_data/nativePAGE/gels/PAGE Organization Document.docx]

12/12/2025

12/16/2025

12/17/2025

01/21/2026
